# *ESR1* Amplification in Breast Cancer by Optimized RNase FISH: Frequent but Low-Level and Heterogeneous

**DOI:** 10.1371/journal.pone.0084189

**Published:** 2013-12-18

**Authors:** Cathy B. Moelans, Frederik Holst, Olaf Hellwinkel, Ronald Simon, Paul J. van Diest

**Affiliations:** 1 Department of Pathology, University Medical Center Utrecht, Utrecht, The Netherlands; 2 Section of Gynecology and Obstetrics, Department of Clinical Science, Haukeland University Hospital, Bergen, Norway; 3 Department of Legal Medicine, University Medical Center Hamburg Eppendorf, Hamburg, Germany; 4 Department of Pathology, University Medical Center Hamburg Eppendorf, Hamburg, Germany; The University of Hong Kong, China

## Abstract

Prevalence of *ESR1* amplification in breast cancer is highly disputed and discrepancies have been related to different technical protocols and different scoring approaches. In addition, pre-mRNA artifacts have been proposed to influence outcome of *ESR1* FISH analysis. We analyzed *ESR1* gene copy number status combining an improved RNase FISH protocol with multiplex ligation-dependent probe amplification (MLPA) after laser microdissection. FISH showed a high prevalence of *ESR1* gains and amplifications despite RNase treatment but MLPA did not confirm *ESR1* copy number increases detected by FISH in more than half of cases. We suggest that the combination of the *ESR1*-specific intra-tumor heterogeneity and low-level copy number increase accounts for these discrepancies.

## Introduction

Since the discovery of trastuzumab for treatment of *HER2*-amplified breast cancer, fluorescence in-situ hybridization (FISH) based gene copy number analysis has become a standard tool in molecular pathology laboratories. While *HER2* amplification is usually high-level, other clinically relevant genes may show only low-level copy number alterations, e.g. *EGFR*, *HER3*, and *PIK3CA* gene copy number alterations in lung cancer [1-11]. The *ESR1* gene, encoding estrogen receptor alpha (ER), is probably the most disputed example of such low-level amplified genes. Since our initial report of 36% copy number increase [12], numerous studies have either confirmed [13] or challenged [14] that *ESR1* is frequently gained in breast cancer. Discrepancies have been related to different copy number enumeration methods and scoring criteria while sensitivity challenges of non morphological methods using isolated DNA are still not fully addressed [15,16]. Further it was suggested that hybridization of the FISH probe to ESR1 pre-messenger RNA could result in aggregates of FISH signals which might be easily misinterpreted as gene amplification [17]. However, RNase treatment can impair FISH analysis by its DNA binding properties [18,19]. To estimate the influence of RNase treatment on *ESR1* FISH analysis outcome, we developed an improved RNase FISH protocol permitting complete RNA elimination after pepsin digestion including a thorough RNase removal and, thus, a clean and clear gene copy number visualization. We analyzed breast cancer large sections and tissue microarrays (TMAs) using standard and RNase FISH protocols, and also employed multiplex ligation-dependent probe amplification (MLPA) after laser-capture microdissection of tumor cells as an RNA-independent means of *ESR1* copy number determination. Large sections were taken from 24 high-grade breast cancers with *ESR1* amplification according to pre-existing FISH results from a “classical” FISH assay and a differential qRT-PCR approach using *SOD2* and *ESR2* as reference genes [12]. *ESR1* copy numbers were re-assessed in 1-4 different cancerous areas in each of these 24 tumors (total: 50 areas) by a commercial FISH assay resulting in 15 tumors showing *ESR1* amplification and 9 showing *ESR1* gain, followed by a validation by MLPA and our improved RNase FISH analysis. In addition, a TMA of 147 consecutive breast cancers was analyzed for validation with the classical and the RNase FISH protocol.

## Methods

### Ethics statement

All tissue samples included in this study were double-pseudomized left-over samples from pathological routine diagnosis in the Department of Pathology, University Medical Center Hamburg Eppendorf, Hamburg, Germany, that can be used for research without informed consent according to the laws of the State of Hamburg, Germany (§12 HmbKHG). Manufacturing and usage of tissue microarrays for research purposes has been approved by the local Institutional Review Board (WF-049/09) of the Aerztekammer Hamburg (Chair: Prof. T. Weber).

### Patient material

Out of a consecutive subset of 90 formalin fixed, paraffin embedded grade 3 breast cancers from the archives of the Department of Pathology in Hamburg that had been previously used in an *ESR1* FISH mapping study [12], a total of 35 cases were selected including 24 tumors with increased *ESR1* copy numbers by FISH (15 amplified and 9 gained) and 11 tumors without *ESR1* copy number increase. In most cases more than one tissue area was selected for FISH/MLPA analysis, resulting in all together 50 different tumor areas with and 19 areas without *ESR1* copy number increase. 13 of the 24 tumors with and 10 of the 14 tumors without increased *ESR1* copy number by FISH had previously been examined by TaqMan RT-qPCR using *ESR2* and *SOD2* as reference genes (primer and probe sequences see Table S3, method as previously described [12]). In addition, a tissue microarray (TMA) was constructed from 147 consecutive - but selected for availability of at least 8 tumor containing tissue blocks - breast tumors (14% grade 1, 57% grade 2 and 29% grade 3) and subjected to FISH analysis. 

### FISH

Standard *ESR1* FISH analysis was performed using the ZytoLight®SPEC ESR1/CEN 6 Dual Color Probe Kit (Zytovision, Germany, Z-2070-20) according to the manufacturer’s instructions with minor modifications. Briefly, slides were deparaffinized and incubated for 15 minutes in Heat Pretreatment Solution Citric at 98°C. Slides were incubated in a pepsin solution for 10 min at 37° C, washed in Wash buffer SSC, dehydrated and air dried. Subsequently, 10 μl of ZytoLight®SPEC ESR1/CEN 6 Dual Color Probe was applied to the slides followed by denaturation at 75° C for 10 min and incubation for 48-72 hours at 37° C in a Thermobrite StatSpin system (Abbott Molecular). After hybridization, coverslips were removed in Wash Buffer A at 37° C for 2 min, followed by wash steps in the same Wash Buffer for 2× 5 min at 37° C, dehydration. Slides were counterstained with DAPI/Antifade solution for 15 min in the dark. 

For FISH with RNase pretreatment, an additional RNase digestion step was introduced into the standard protocol after pepsin treatment (providing a better access of RNase to the gene locus than before pepsin digestion). Slides were rinsed in 2× SSC buffer and incubated with RNase A solution (100µg / ml in 2× SSC, pH 7.4, Macherey-Nagel) for 30 min at 37°C. Due to the binding affinity of RNase A to (especially denatured single stranded) DNA, the DNA-bound enzyme blocks FISH probe binding to the target DNA and FISH signals are weakened or totally undetectable. To resolve the DNA-RNase complex an additional NaCl washing step was performed after RNase treatment [18,19]. Slides were rinsed 5 min in 2× SSC followed by 5 min washing in 0.1 M NaCl at room temperature, before the standard protocol was continued. Without the 0.1 M NaCl washing step, interpretation of FISH results was hindered due to lack of signals of sufficient brightness and quality.

A strip protocol was used [20] to analyze the same tissue sections twice both with and without RNase treatment. Wash buffer (2× SSC, 0.3 % NP 40) was added to release coverslips and remove DAPI/glycerol mix and oil residues. After a short 2× SSC wash, slides were incubated with denaturation solution (70% formamide / 2× SSC, pH 7.0-7.4) at 73°C for 2 × 2.5 minutes to remove the hybridized probe. This was followed by a short 2× SSC washing step, incubation with RNase A and a NaCl washing step, according to the modified standard protocol. For EGFR FISH, a probe combination of EGFR (LSI EGFR SpectrumOrange, Vysis/Abbott) and centromere 7 (CEP 7 SpectrumGreen, Abbott) was used as previously described [11]. RNase A digestion consistently resulted in the disappearing of fuzzy signal clouds (like pre-mRNA appears [21-23]), while sharp point shaped signals (clusters) remained. This effect was interpreted as the different appearance of pre-mRNA (fuzzy clouds) and DNA copy (point-shaped signals) mediated FISH signals due to pre-mRNA processing [24], and was taken as an internal control for complete RNA elimination. Evaluation of FISH copy numbers was carried out using a Zeiss Axio Imager A1 fluorescence microscope equipped with a Zeiss AxioCam and AxioVision imaging software. Analyzed whole sections and TMA spots where attentively manually scanned for copy number elevations. Copy number was determined by taking the full z-axis size of the analyzed nuclei into account [25]. In case of tumor heterogeneity, within the tumor region with the highest copy number increase by FISH, 20 nuclei were randomly selected for copy number determination and used to define the amplification status of the according area. Interpretation was based on the average copy number ratio *ESR1*/*CEN6* in 20 entire and non-overlapping nuclei. Applying FISH analysis without RNase treatment in tumors with cell nuclei showing tight as well as confluent signal clusters, the copy number was determined in nuclei with distinguishable signals. An average ratio ≥2 was rated as amplification, and ≥1.3 as gain [26]. Interpretation of FISH results after RNase treatment was blinded to FISH results without RNase treatment. Cases without an elevated copy number (ratio *ESR1*/*CEN6* ≥1.3) including normal (ratio *ESR1*/*CEN6* = 2/2), low level gained (ratio *ESR1*/*CEN6* <1.3), deleted and “polysomic” cases, were defined as “not increased”.

### Laser microdissection

Laser microdissection (PALM, Zeiss) was performed on 4 to six 3 µm thick paraffin sections (by comparing with a serial H&E stained slide where FISH amplified/non-amplified areas were marked). For laser microdissection, sections were baked at 56°C for 1 hour, deparaffinized in xylene for 10 minutes and rehydrated through graded alcohols (100%, 85% and 70% for 1 minute each). After staining with haematoxylin for 5 seconds, slides were rinsed in water and dipped in eosin for 5 seconds. Finally, slides were dehydrated in 100% ethanol for 1 minute and air dried.

### Multiplex ligation-dependent Probe Amplification (MLPA)

After laser microdissection, DNA was isolated by overnight incubation in proteinase K (10 mg/ml; Roche, Almere, The Netherlands) at 56°C followed by heat inactivation for 10 min. The DNA supernatant (30-50 μl) was, after centrifugation, used in the MLPA analysis according the manufacturers' instructions, using the P078-B1 breast kit (MRC Holland, Amsterdam, The Netherlands) containing two *ESR1* probes. All tests were performed in duplicate on an ABI 9700 PCR machine (Applied Biosystems, Foster City, CA, USA). PCR products were analysed on an ABI3730 DNA analyser (Applied Biosystems). Gene copy numbers were analyzed using Genemapper (Applied Biosystems) and Coffalyser (version 7.0) software (MRC-Holland). Six negative reference samples (4 blood samples and 2 normal breast samples) were taken along in each MLPA run to normalize MLPA ratios.

To avoid loss of sensitivity due to suboptimal probe sequence specific properties, results of both probes where compared by calculating the average MLPA ratio for each of the two MLPA *ESR1* probes, based on 19 tumor areas showing no copy number increase by FISH (average *ESR1* copy number: 1.95, average *ESR1*/*CEP6* ratio: 0.99). The first probe showed an average MLPA copy number ratio of 1.01 and thus an almost exact normalization whereas the second MLPA probe showed a ratio of only 0.83 indicating a less efficient amplification leading to a significant difference in copy number ratio between both probes (*p*<0.001). Therefore, only the first MLPA probe was used for further calculations and comparisons. An MLPA ratio value below 0.7 was defined as loss, a value between 0.7-1.3 was defined as normal, 1.3-2.0 as gain, and values >2.0 were defined as (high level) amplification, as established previously [27-30]. Interpretation of MLPA was done blinded to FISH results. Positive MLPA results (ratio ≥1.3) were matched to previously existing FISH mapping data [13]: 8 (of 16) cases with MLPA copy number elevations were predominantly limited to the *ESR1* gene only whereas 6 (of 8) cases showed more extended amplicons, (see Table S1).

### Statistics

Statistics were performed using SPSS statistical software (15.0). ER en PR protein status were determined according to Remmele et al. [31], with a score >2 regarded as positive. Differences in FISH, MLPA or qPCR copy number ratios were evaluated using the Mann-Whitney U test (unpaired) or Wilcoxon signed rank test (paired). Correlation between FISH and MLPA was calculated using the Spearman rho correlation coefficient. Two-sided p-values <0.05 were defined as significant. Figures were made using GraphPad Prism 5.

## Results

### Effect of RNase treatment on FISH copy numbers and signal size and shape

**Figure 1:**
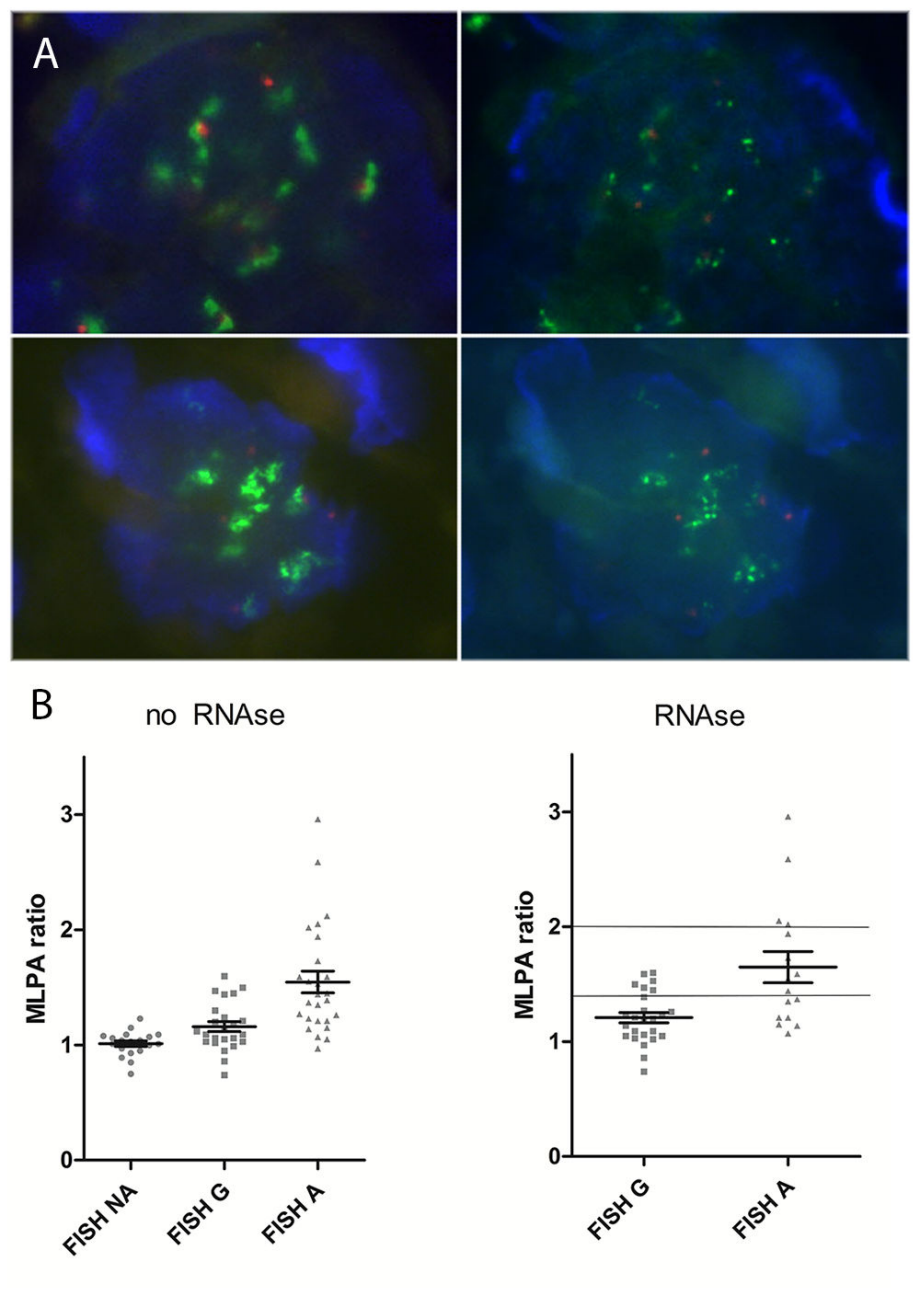
Effect of RNase treatment on FISH and on correlation between FISH and MLPA. (a) RNase pretreatment resulted in a higher fraction of tumor cells showing point-shaped FISH signals, by eliminating eye catching fuzzy clusters of ESR1 signals seen by standard FISH (b) MLPA copy number ratios in FISH “not increased”, gained and amplified samples without and with RNase treatment.

RNase pretreatment resulted in a higher fraction of tumor cells showing point-shaped FISH signals, by eliminating fuzzy clusters (fringes, tails or clouds) of *ESR1* FISH probe signals (Figure 1) seen in many nuclei by standard FISH. 

But FISH with and without RNase treatment showed no major differences in gene copy number, neither in the whole sections, nor in the TMA format. In the 50 whole section areas of 24 breast carcinomas with increased copy number, the average *ESR1* copy number was 4.55 (range 2.30-12.05) pre- and 4.44 (range: 2.10-13.70) post-RNase (*p*=0.47) and the average *ESR1*/*CEP6* ratio was 2.16 (range 1.28-6.89) pre- and 1.98 (range 1.13-5.37) post-RNase (*p*=0.006). Accordingly, the amplification status remained unchanged in 12/15 tumors (cut-off ratio 2.0) after RNase treatment, whereas three cases were down-staged to gains (cut-off ratio 1.3). 7/9 FISH-gained cases remained gained after RNase treatment, one case was reclassified as amplified and the other as not increased (cut-off ratio 1.3). To illustrate signal appearance, all 24 cases with whole sections are documented with and without RNase treatment in a supplementary photographical dataset (Optical Dataset S1 and S2) 

In the TMA, 82/147 tumor spots were analyzable by classical and RNase FISH. Without RNase, 32 (39%) tumors showed an increased *ESR1* copy number including 11 (13%) amplifications and 21 (26%) gains. After RNase treatment, 30 (37%) tumors showed increased *ESR1* copy numbers (13 (16%) amplifications and 17 (21%) gains), while the *ESR1* status changed from “gain” to “not increased” in two, from “amplification” to “gain” in one, and from “gain” to “amplification” in three tumors (Table S2). The average *ESR1* copy number for the 32 cases considered “*ESR1* copy number increased” pre-RNase was 3.39 pre- and 3.49 post-RNase (*p*=0.558), and the average *ESR1*/*CEP6* ratio was 1.90 (range 1.31-3.08) pre- and 2.05 (range 1.15-4.16) post-RNase (*p*=0.83).

Taken together, in 94.6% of tumors with increased *ESR1* gene copy number before RNase treatment, the *ESR1* status remained increased (mostly cluster type including gene dupli- and triplication [9,32,33]) after RNase treatment. Interestingly, a commercially available *EGFR* FISH probe (Abbott) showed the same RNase sensitive changes of signal appearance as the *ESR1* probe in this study (see Figure S1).

### Effect of RNase treatment on correlation between FISH and MLPA

Tumors with and without *ESR1* copy number increase by FISH showed significant differences by MLPA and qPCR regardless of the assay used (qPCR *ESR1*/*ESR2 p*= 0.002; qPCR *ESR1*/*SOD2 p*= 0.010; MLPA *p*=0.011), although qPCR ratios after *ESR2* normalization were significantly higher than after *SOD2* normalization (*p*<0.0001).

MLPA ratios were highly correlated with FISH ratios regardless of whether FISH was performed with or without RNase pretreatment. Tumors that were rated gained or amplified by FISH had significantly higher MLPA ratios than those with “not increased” FISH findings (*p*=0.0005 for gain and *p*=0.0125 for amplification without RNase; *p*<0.0001 for gain and *p*=0.0041 for amplification with RNase; Figure 1). Spearman rho correlation between MLPA and FISH in cases with elevated copy numbers (gains and amplifications) by FISH, prior and after RNase treatment, was 0.57 (0.35-0.73) and 0.56 (0.31-0.74), respectively (both *p*<0.0001). The overall correlation between FISH with and without RNase was 0.78 (0.62-0.88; *p*<0.0001) (see Figure S2). 54% (13/24) of FISH-increased tumors without RNase treatment, and 57% (13/23) of FISH-increased tumors after RNase treatment failed to yield increased copy numbers by MLPA. Although the fraction of “MLPA failures” was highest (7/10) for tumors with gains by FISH, there were also 3/13 tumors with apparently homogeneous and high-level FISH amplification (for example case 7 and 16 from Table S1) that failed to show an *ESR1* copy number increase by MLPA. FISH positive but MLPA negative samples had a significantly lower FISH copy numbers than MLPA positive samples (1.79 *vs.* 2.37 after RNase; *p*=0.009), as well as a higher rate of heterogeneity (83% (19 of 23 cases) *vs.* 41% (7 of 17 cases); *p*=0.007), and averaged a significantly higher MLPA ratio compared to FISH negative cases (1.11 *vs.* 1.01, *p*=0.015) (Figure 1). 

### Heterogeneity

To take influence of heterogeneity into account, 70 different areas selectively identified by FISH analysis from 20 cancers were microdissected and subjected to MLPA analysis. As illustrated in Table S1, MLPA yielded considerably heterogeneous results from different areas of the same tumor block as well as between different tumor blocks of the same cancer specimen. Of all cases with multiple tumor blocks and/or areas analyzed, 25% (5/20) showed a different copy number status by MLPA between or within blocks, and 39% (7/18) and 67% (8/12) by FISH without and with RNase, respectively. The average copy number ratio variation between/within tumor blocks was 0.55/0.56 for FISH +/- RNase, and 0.25 for MLPA. Furthermore, particularly challenging for copy number determination in tumors, mosaic heterogeneity (cell-to-cell variation)- a well-known phenomenon for many genes [34-36] - was frequently (60% of tumors in our study) observed, mostly in case of low copy number levels. 

## Discussion

In tumors with increased *ESR1* copy number previously determined by a classical FISH assay, RNase A digestion prior to *ESR1* FISH eliminated cloudy signal clusters caused by pre-mRNA artifacts, resulting in clearly distinguishable signals, mostly of cluster (HSR) type. This phenomenon may not be limited to *ESR1* FISH analysis, since similar artifacts were seen also in a case of *EGFR*-amplified lung cancer. These RNA-induced artifacts may pose a problem in particular for the interpretation of low-level amplified genes including *ESR1*, where only few extra gene copies determine the amplification status. However, in our study, RNase digestion did not significantly change the observed *ESR1* gene copy number results. Overall, the fraction of tumors with elevated *ESR1* copy numbers changed only slightly from 39% to 37%. Especially as FISH analysis is the subjective interpretative translation of optical patterns into numerical information, these data suggest that the inter- and intra-observer differences on *ESR1* copy number assessment and different analysis approaches (such as z-stack layers taken into account [25,37], number of countable nuclei considered sufficient for analyzability [38], selected tissue areas chosen, cut-offs used for status definition [17,26,37,38] or even accepted signal quality) may be more influential than removal of pre-messenger RNA itself. In fact, in our hands, RNase pretreatment resulted in a higher fraction of tumor cells showing point-shaped FISH signals, by prohibiting eye catching pre-mRNA artifacts (Figure 1) seen in many nuclei by standard FISH, in line with the observations of Ooi. et al [17]. Nevertheless, it was possible to reliably determine *ESR1* copy numbers in all large sections also without RNase treatment, since sufficient numbers of nuclei showed distinct and countable signals (see Optical Dataset S1 and S2). 

While Ooi et al. reported a gain frequency of 5.9% (3/51) in breast carcinoma using FISH after RNase pre-treatment, the findings of the present study give evidence for copy number increase in 37% of cases. This large difference could be related to several factors. First, in contrast to Ooi et al [23] (personal communication), we used a different RNase treatment protocol where RNase digestion was performed after pepsin digestion and an additional NaCl step was introduced to eliminate DNA bound RNase. In addition we used a different FISH assay. Both could have significant influence on signal appearance. Second, we used full section tissue slides and a TMA (FFPE) instead of core needle biopsies (FFPE) and touch smears, and a different (subjective) way of analysis. For example, Ooi et al. used the HER2 ASCO criteria for evaluation of *ESR1* (1.8 and 2.2 cut-offs) whereas we used 1.3 and 2.0 as cut-off. Lastly, the choice of study population may also be of significant influence, as e.g. in early breast cancer *ESR1* amplification was detected at a lower frequency (14% of ER positive cases in BIG 1-98 trial) [39] compared to advanced stage breast cancer (23% revealed by the same FISH technique) [26].

Independent from RNase treatment, there was a significant difference in MLPA copy number ratio between samples that were FISH amplified and samples that showed only gain or no *ESR1* copy number increase, although MLPA did not confirm *ESR1* copy number increases detected by FISH in more than half of cases. Nevertheless this discrepancy was independent from RNase treatment, suggesting that reasons other than RNA-related hybridization artifacts account for the differences in *ESR1* copy number alterations observed with different methods.

This finding either suggests false-positive FISH findings independently from RNase treatment, or, and probably more likely, that MLPA is not capable of identifying all tumors with increased *ESR1* copy numbers due to threshold and dilution issues. As expected, different areas of one tumor showed different copy numbers by FISH as well as by MLPA. Of all tumors with multiple areas analyzed, 25% showed a different copy number status by MLPA between areas, and 39% and 67% by FISH without and with RNase, respectively. Revealingly, the FISH positive but MLPA negative samples had a significantly lower FISH copy number than MLPA positive cases and averaged a significantly higher MLPA ratio compared to FISH negative cases. Furthermore, FISH positive but MLPA negative samples had a higher rate of heterogeneity.

Since all tumor samples had been laser capture microdissected to minimize the influence of contaminating non-neoplastic cells, we therefore hypothesize that the low-level of *ESR1* copy number changes and copy number heterogeneity could have obscured detection of *ESR1* amplification by MLPA (e.g. in samples 5, 9 and 26, see Table S1).

Furthermore different efficiency of probe amplification (average MLPA *ESR1*/*CEP6* ratio of 0.99 vs 0.83) and qPCR assay ratios (*p*<0.0001) indicate that probe design and normalization may significantly influence the results of such DNA quantifying methods, which is especially important in case of values around the threshold.

In summary these observations provide a probable explanation for the discrepant results that have been reported by *ESR1* copy number assays using isolated DNA (such as MLPA), FISH and CISH.

## Supporting Information

Figure S1 **A commercially available EGFR FISH probe (Abbott) showed the same RNase sensitive changes as the ESR1 probe in this study.**
(JPG)

Figure S2 **Correlation between MLPA and FISH.**
(JPG)

Table S1 **MLPA results and FISH results without and with RNase treatment, performed on 35 breast tumors.**
(XLSX)

Table S2 **FISH results without and with RNase treatment, performed on a breast TMA.**
(XLSX)

Table S3 **TaqMan RT-qPCR Primer and Reporter Sequences.**
(DOC)

Optical Dataset S1 **Optical dataset illustrating FISH signal appearance containing full size *ESR1* FISH photos (not all of them representative) of a total of 35 breast cancer cases (Case#1-35) including 24 tumours with increased ESR1 copy numbers by FISH (15 *ESR1* amplified and 9 *ESR1* gained, Case#01-#24) and 11 tumours without *ESR1* copy number increase (Case#25-#35), selected out of a consecutive subset of 90 formalin fixed, paraffin embedded grade 3 breast cancers from the archives of the Department of Pathology in Hamburg that had been previously used in an *ESR1* FISH mapping study [12].** For all 24 cases with *ESR1* copy number increase different pictures (Fig.01- …) **without RNase A treatment** are available. The pictures document by three color photographs the *ESR1* FISH appearance of nuclei with *ESR1* copy number increase and nuclei without *ESR1* copy number increase on 4µm full section FFPE tissue slides, showing *ESR1* signals (green), *CEN6* signals (orange) and nuclei (blue) in 100x or 63x magnification. Pictures are subscribed “CNI” for observed gene “copy number increase” and “NO” for “normal” or “no copy number increase”. Gene loci with additional allelic copies (CNI) are marked exemplarily in some cases (white arrows and edges). Especially if nuclei with “CNI” occur in a pattern of mosaic heterogeneity intermingled with nuclei without “CNI”. Due to intensity variations and the three dimensional distribution of signals not all present gene copies can be shown. To illustrate the distribution of signals within the z-axis, some pictures are taken with two different z-layers (Z-Stack A-D). To illustrate the difference between cases (with tissue areas) showing *ESR1* copy number increase (CNI) and cases without *ESR1* copy number increase, pictures of the 11 cases with clearly “normal” copy number or “no copy number increase” (“NO”) are shown in addition.
(DOCX)

Optical Dataset S2 **Optical dataset illustrating FISH signal appearance containing full size *ESR1* FISH photos (not all of them representative) of a total of 35 breast cancer cases (Case#1-35) including 24 tumours with increased ESR1 copy numbers by FISH (15 *ESR1* amplified and 9 *ESR1* gained, Case#01-#24) and 11 tumours without *ESR1* copy number increase (Case#25-#35), selected out of a consecutive subset of 90 formalin fixed, paraffin embedded grade 3 breast cancers from the archives of the Department of Pathology in Hamburg that had been previously used in an *ESR1* FISH mapping study [12].** For all 24 cases with *ESR1* copy number increase different pictures (Fig.01- …) with RNase A treatment are available. The pictures document by three color photographs the *ESR1* FISH appearance of nuclei with *ESR1* copy number increase and nuclei without *ESR1* copy number increase on 4µm full section FFPE tissue slides, showing *ESR1* signals (green), *CEN6* signals (orange) and nuclei (blue) in 100x or 63x magnification. Pictures are subscribed “CNI” for observed gene “copy number increase” and “NO” for “normal” or “no copy number increase”. Gene loci with additional allelic copies (CNI) are marked exemplarily in some cases (white arrows and edges). Especially if nuclei with “CNI” occur in a pattern of mosaic heterogeneity intermingled with nuclei without “CNI”. Due to intensity variations and the three dimensional distribution of signals not all present gene copies can be shown. To illustrate the distribution of signals within the z-axis, some pictures are taken with two different z-layers (Z-Stack A-D). To illustrate the difference between cases (with tissue areas) showing *ESR1* copy number increase (CNI) and cases without *ESR1* copy number increase, pictures of the 11 cases with clearly “normal” copy number or “no copy number increase” (“NO”) are shown in addition.
(DOCX)
