# Correction: *ESR1* Amplification in Breast Cancer by Optimized RNase FISH: Frequent but Low-Level and Heterogeneous

**DOI:** 10.1371/annotation/d9c3406a-75d0-471c-80a7-8ed4cff275bc

**Published:** 2014-01-23

**Authors:** Cathy B. Moelans, Frederik Holst, Olaf Hellwinkel, Ronald Simon, Paul J. van Diest

The Competing Interests statement is incorrect. The correct Competing Interests statement is: The University Medical Centre Hamburg-Eppendorf has filed patent US8101352B2 “Detection of ESR1 Amplification in Breast Cancer” and accordant patent applications in EU and CA for certain technology described in this paper.

