# Supplementary figures and images for: ESR1 Amplification in Breast Cancer by Optimized RNase FISH: Frequent but Low-Level and Heterogeneous
Source: PLoS One. 2013 Dec 18;8(12):e84189. doi: 10.1371/journal.pone.0084189 (PMC3867473; doi:10.1371/journal.pone.0084189)

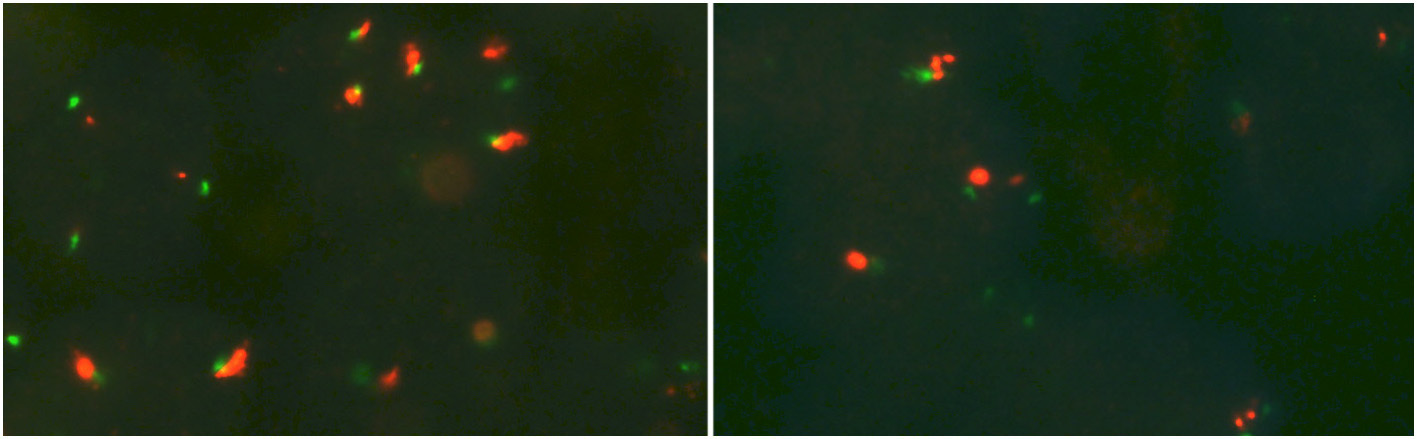

Supplement: Figure S1 — A commercially available EGFR FISH probe (Abbott) showed the same RNase sensitive changes as the ESR1 probe in this study. (JPG) [file pone.0084189.s001.jpg]

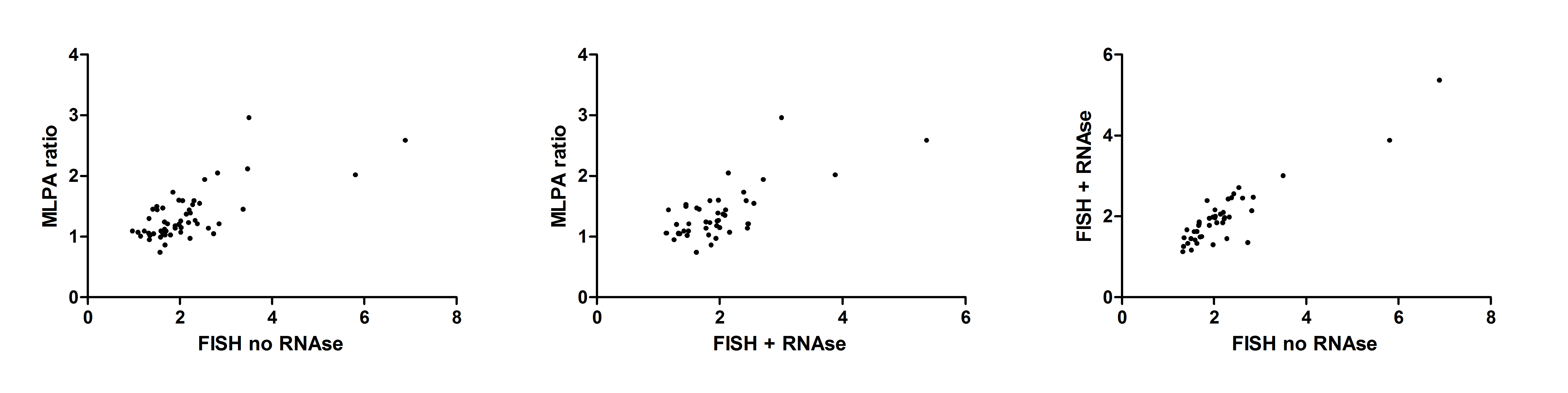

Supplement: Figure S2 — Correlation between MLPA and FISH. (JPG) [file pone.0084189.s002.jpg]
