# Supplementary material for: ESR1 Amplification in Breast Cancer by Optimized RNase FISH: Frequent but Low-Level and Heterogeneous
Source: PLoS One. 2013 Dec 18;8(12):e84189. doi: 10.1371/journal.pone.0084189 (PMC3867473; doi:10.1371/journal.pone.0084189)
Supplement: Table S3 — TaqMan RT-qPCR Primer and Reporter Sequences. (DOC) [file pone.0084189.s005.doc]

**Supplementary Table 3: TaqMan RT-qPCR Primer and Reporter Sequences**

Primer Sequences:

| Forward Primer  Name | Forward Primer Seq. | Reverse Primer  Name | Reverse Primer Seq. |
| --- | --- | --- | --- |
| gESR1-EX1-f | GCCAACGCGCAGGTCTA | gESR1-EX1-r | CGCCGCAGCCTCAGA |
| gESR2-EX5-f | CGGCAAGGCCAAGAGAAGT | gESR2-EX5-r | AGGAGGGTGAGCACTAGCT |
| gSOD2-f | GGTGTCCAAGGCTCAGGTT | gSOD2-r | AATTTGTAAGTGTCCCCGTTCCTT |

Reporter Sequences:

| Reporter Name | Reporter Dye | Reporter Sequence | Reporter Quencher | Reporter type | Design Strand |
| --- | --- | --- | --- | --- | --- |
| gESR1-EX1-p | FAM | CTCCCCTACGGCCCC | NFQ | MGB | Forward |
| gESR2-EX5-p | FAM | CTGGACGCCCTGAGCC | NFQ | MGB | Forward |
| gSOD2-p | FAM | ACCAAGCCAACCCC | NFQ | MGB | Reverse |
