# Supplementary material for: ESR1 Amplification in Breast Cancer by Optimized RNase FISH: Frequent but Low-Level and Heterogeneous
Source: PLoS One. 2013 Dec 18;8(12):e84189. doi: 10.1371/journal.pone.0084189 (PMC3867473; doi:10.1371/journal.pone.0084189)
Supplement: Optical Dataset S2 — Optical dataset illustrating FISH signal appearance containing full size ESR1 FISH photos (not all of them representative) of a total of 35 breast cancer cases (Case#1-35) including 24 tumours with increased ESR1 copy numbers by FISH (15 ESR1 amplified and 9 ESR1 gained, Case#01-#24) and 11 tumours without ESR1 copy number increase (Case#25-#35), selected out of a consecutive subset of 90 formalin fixed, paraffin embedded grade 3 breast cancers from the archives of the Department of Pathology in Hamburg that had been previously used in an ESR1 FISH mapping study [12]. For all 24 cases with ESR1 copy number increase different pictures (Fig.01- …) with RNase A treatment are available. The pictures document by three color photographs the ESR1 FISH appearance of nuclei with ESR1 copy number increase and nuclei without ESR1 copy number increase on 4µm full section FFPE tissue slides, showing ESR1 signals (green), CEN6 signals (orange) and nuclei (blue) in 100x or 63x magnification. Pictures are subscribed “CNI” for observed gene “copy number increase” and “NO” for “normal” or “no copy number increase”. Gene loci with additional allelic copies (CNI) are marked exemplarily in some cases (white arrows and edges). Especially if nuclei with “CNI” occur in a pattern of mosaic heterogeneity intermingled with nuclei without “CNI”. Due to intensity variations and the three dimensional distribution of signals not all present gene copies can be shown. To illustrate the distribution of signals within the z-axis, some pictures are taken with two different z-layers (Z-Stack A-D). To illustrate the difference between cases (with tissue areas) showing ESR1 copy number increase (CNI) and cases without ESR1 copy number increase, pictures of the 11 cases with clearly “normal” copy number or “no copy number increase” (“NO”) are shown in addition. (DOCX) [file pone.0084189.s007.docx]

**Optical Dataset S2**

<http://dx.doi.org/10.6084/m9.figshare.864525>
